# The Education of the Deaf and Dumb

**Published:** 1897-01-16

**Authors:** 


### THE EDUCATION OF THE DEAF AND DUMB.

In view of recent developments, promised and accomplished, in the teaching of deaf mutes, a report lately issued by a sub-committee of directors of the Glasgow Institution for the Education of the Deaf and Dumb on the methods available for the education of these unfortunates is of the greatest interest and importance. The great question is as to the efficacy of the oral system, as an aid to mental development, and as a means of inter-communication between themselves and others. Much has been said and written of recent years in regard to the advantages of the oral system of teaching deaf mutes. It has been claimed that by this method —by teaching children to articulate, and by training them to understand the articulation of others, the so-called lip reading—deaf and dumb children could be put on level with ordinary children; at any rate, so far that they should be able to understand the conversation of hearing people, and to make themselves understood without the use of signs. There can be no doubt that in certain cases the claim is justified by the results obtained. The question is, How frequently are such results obtained, and when obtained, in how large a proportion of the cases do the pupils, after they have left school, find that they can trust to pure oral methods of communication? Do they tend, when free from control, to drift into those systems which depend on the use of signs and manual alphabets? There is something exceedingly attractive about the oral system. When it is really successful it seems to restore to the pupil the lost faculties both of hearing and of speech. But is it often successful? The answer to this question is most disheartening. The committee visited many schools, both where the pure oral system was taught and where while it was taught in the schools, the sign manual system was permitted among the children during play times; but so far as concerned the power of understanding people with whose modes of speech they were not acquainted, and of making themselves intelligible to strangers, the results were certainly not such as to encourage the exclusive use of this system.

The institutions visited were those at Manchester, Derby, Boston Spa, Margate, Ealing, and Donaldson's Hospital, Edinburgh, and the classes were those conducted by the School Boards of London, Leicester, Nottingham, and Leeds. "In the greater number of classes the articulation, even of advanced pupils, when reading was so indistinct that it was hardly possible to make out more than a word or two here and there. The reading of a few could be followed with some fair measure of success, and in one or two instances it was possible to understand nearly every word. In one class, composed of children who had been taught first on the manual and later on the oral system, the results were remarkably good. With two or three of the pupils of this class conversation was quite practicable, and by the class generally the dictation given by the teacher was well done. Such instances, however, your committee are compelled to say, were by no means common. It should also be mentioned that in some cases the children who showed excellent results had not been born deaf."

To test the practical utility of the oral method as a means for the inter-communication of ideas, the question was put to a considerable number of teachers and others who were known to have had a large experience in connection with deaf mutes, "Can you give instances of orally-taught deaf people profiting by the ordinary religious ordinances for hearing people, or do they resort generally to missions where the sign and manual system prevails?"

The answers received to this question were all to the effect that even the best lip readers cannot so follow and understand an ordinary sermon, and that nearly all resort to sign and manual services.

Even as regards communication between each other it seems plain that the tendency, so soon as they are free, is to resort to the manual alphabet.

As to obtaining employment, it seems that the masters who employ deaf work-people express no preference for the orally-taught, and that wherever accuracy is of importance resort is had to writing, whatever method of teaching has been adopted. Nor need we wonder at this when we see the answers to the question "Have you ever met one or more orally-taught deaf mutes with whom you could freely converse?" To this ten gave a direct negative, four a simple affirmative, while amonget the others were such answers as these: "Yes; perhaps nine." "Cannot recall any case in which the conversation was not laboured." "Many, when the conversation was about commonplaces." "Not freely, but with care and pains had conversed with many." Now when we compare these answers with what one sees every day in the streets—deaf people chattering away with the greatest freedom by aid of the sign-manual system as they walk along—we cannot but fear that in practice the oral system has not turned out as well as it was hoped would be the case.

It has been too readily assumed by the Oralists that the faculty of speech, on which intellectual development so much depends, means oral speech alone. If this were so, if it were the only key to mental progress, no doubt it would be worth while to spend weary years in teaching children to talk, however poor the result might be. But we have ample proof that as an aid to mental processes that form of speech is the best which in the readiest manner enables the speaker to register ideas and to communicate with others, and when we find it said of those orally taught that "if they understand signs will use them in ninety-nine cases out of a hundred" we cannot admit, without further evidence, that the oral method is the best in cases where the hearing has been entirely lost. Where some hearing is left, and especially where a child has learned to talk a little before becoming deaf the matter is different.

